# Physicochemical Characterization and Pharmacological Evaluation of Novel Propofol Micelles with Low-Lipid and Low-Free Propofol

**DOI:** 10.3390/pharmaceutics14020414

**Published:** 2022-02-14

**Authors:** Yongchao Chu, Tao Sun, Zichen Xie, Keyu Sun, Chen Jiang

**Affiliations:** Department of Pharmaceutics, School of Pharmacy, Key Laboratory of Smart Drug Delivery (Ministry of Education), Minhang Hospital, State Key Laboratory of Medical Neurobiology, MOE Frontiers Center for Brain Science, Research Center on Aging and Medicine, Fudan University, Shanghai 201203, China; ytuyongchao@163.com (Y.C.); 18918169503@163.com (Z.X.)

**Keywords:** propofol, micelle, DSPE mPEG2k, Solutol HS 15

## Abstract

We developed safe and stable mixed polymeric micelles with low lipids and free propofol for intravenous administration, to overcome the biological barrier of the reticuloendothelial system (RES), reduce pain upon injection, and complications of marketed propofol formulation. The propofol-mixed micelles were composed of distearoyl-phosphatidylethanolamine-methoxy-poly (ethylene glycol 2000) (DSPE mPEG2k) and Solutol HS 15 and were optimized using Box Behnken design (BBD). The optimized formulation was evaluated for globule size, zeta potential, loading content, encapsulation efficiency, pain on injection, histological evaluation, hemolysis test, in vivo anesthetic action, and pharmacokinetics, in comparison to the commercialized emulsion Diprivan. The optimized micelle formulation displayed homogenous particle sizes, and the free drug concentration in the micelles was 60.9% lower than that of Diprivan. The paw-lick study demonstrated that propofol-mixed micelles significantly reduced pain symptoms. The anesthetic action of the mixed micelles were similar with the Diprivan. Therefore, we conclude that the novel propofol-mixed micelle reduces injection-site pain and the risk of hyperlipidemia due to the low content of free propofol and low-lipid constituent. It may be a more promising clinical alternative for anesthetic.

## 1. Introduction

Propofol (2,6-diisopropylphenol) is a short-acting sedative and hypnotic agent that is most frequently administered as an intravenous anesthetic in clinics. It exhibits multiple advantages, e.g., rapid onset and recovery times, and a short duration of action [1,2]. Propofol is a highly lipophilic compound with limited solubility in water (154 μg/mL) [3]. Due to its lipophilic properties, propofol can easily penetrate the blood–brain barrier and lead to rapid anesthetic effects. Moreover, propofol displays a “trend”, involving “low accumulation” in the human body, owing to the short half-life in vivo, and rapid clearance [4]. However, due to its poor solubility in water, the development of a novel propofol injectable has become a major challenge with considerable industrial potential and academia significance.

Propofol was initially formulated in a 16% Cremophor EL solvent [5]. However, due to anaphylaxis caused by Cremophor EL, a fat emulsion (Diprivan) was developed by utilizing soybean oil, purified egg lecithin, and glycerol [6]. Despite the considerable success of the present formulation in the market, some defects have been widely reported for the commercial emulsion based on lipids, which limits its clinical application [7]. For instance, intravenous injection of Diprivan often accompanies injection-site pain caused by free propofol. In addition, the fat emulsion of propofol suffers increased chances of hyperlipidemia for prolonged sedation in intensive care unit (ICUs) due to the high lipid content and high risk of external microbial contamination on repeat administration, as a result of high lecithin and soybean oil content [8]. Diprivan containing lecithin is not suitable for vegetarian or religionists. Moreover, due to thermodynamic instability of emulsion, the particle sizes increase significantly during storage. These large droplets might accumulate in the reticuloendothelial system (RES) organs, which could result in increasing oxidative stress and tissue damage to the liver [9].

Researchers have developed multiple approaches to overcome the drawbacks of Diprivan, including use of microemulsions and prodrugs, and complexes with cyclodextrins. Fospropofol, a prodrug of propofol, has been found to reduce pain upon injection compared to propofol. However, the prodrug has a slower onset of efficacy [10]. A novel propofol by complexation with cyclodextrin was also developed, but adverse hemodynamic consequences induced by the complexes limit its further application [11]. Thus, there is an urgent need to develop a novel propofol formulation for-avoiding the undesirable defects of the marketed formulation. In recent years, polymeric micelles have demonstrated enormous potential in improved solubility of poorly water-soluble drugs [12,13,14,15]. In 2019, the FDA approved Cequa, the first micelle-based formulation. Generally, when the critical micellization concentration (CMC) is exceeded, amphipathic copolymers self-assemble into core–shell nanostructures in water [16,17]. The copolymer micelle, as a novel carrier, exhibits many unique advantages, such as lessened undesirable effects of drugs, increased drug loading and it is stable during storage [18,19]. Furthermore, they are able to improve drug stability by protecting the molecules from premature degradation due to the core–shell nanostructure [20,21,22]. Consequently, there has been a need to develop a promising micelle formulation that could effectively resolve the above-mentioned issues.

**Figure 1:**
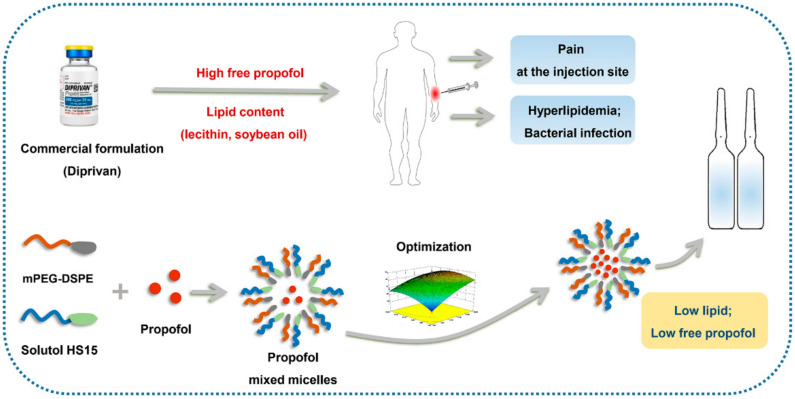
The schematic representation for the propofol mixed micelles.

In order to replace the high level of lipid component and reduce the concentration of free propofol, this study presents a novel micelles formulation based on DSPE mPEG2k and Solutol HS 15. DSPE mPEG2k has emerged as an excellent drug carrier for therapeutics since the initial discovery of its ability to form polymeric micelles in aqueous environments in 1994 [23]. Solutol HS 15 is a potent non-ionic solubilizing agent with low toxicity and a strong solubilization effect [24]. Its relatively bulky hydrophobic compartment possibly facilitates better drug solubilization. The tailored composition of the hydrophobic block can achieve stable encapsulation of lipophilic molecules. A DSPE mPEG2k/Solutol HS 15 mixed micellar structure not only reduces the concentration of free propofol in the aqueous phase, but it also provides a new formulation of propofol with higher safety and efficacy attributes (Figure 1). This study evaluates the new formulation of the propofol-mixed micelle for size distribution, zeta potential, pH, osmolarity, morphology, and the degree of free propofol in the aqueous phase. Moreover, in vivo efficacy and pharmacokinetic studies were conducted in rats.

## 2. Materials and Methods

### 2.1. Materials

Propofol was provided from Shanghai Pharmaceuticals Holding Co., Ltd. (Shanghai, China). DSPE mPEG2k copolymers were purchased from AVT Pharmaceutical Tech Co., Ltd. (Shanghai, China). Solutol HS15 was supplied from BASF (Berlin, Germany). Diprivan was obtained by Astra-Zeneca Ltd. (London, UK). Acetonitrile was chromatography-grade and other solvents were of analytical grade.

### 2.2. Animals

Sprague–Dawley (SD) rats (180–220 g) and BALB/c mice (20–25 g) were supplied from Shanghai SLAC Laboratory Animal Co. Ltd. (Shanghai, China) and All animal handling procedures were approved by Institutional Animal Care and Use Committee of China (2019-03-YJ-JC-01).

### 2.3. Preparation of Propofol-Mixed Micelles

The propofol-mixed micelle formulation was formulated by a film dispersion method [25]. Briefly, a defined amount of propofol, DSPE-mPEG2k, and Solutol HS 15 was dissolved in a methanol solution and evaporated by rotatory evaporator at 37 °C to ensure the formation of homogeneous film. The thin film was hydrated in saline solution (0.9% sodium chloride) at 37 °C and obtained a uniform spherical micellar solution after filtration through a 0.22 μm filter.

### 2.4. Determination of Encapsulation Efficiency and Loading Content

The encapsulation efficiency (EE) of mixed micelles was determined as follows. The micelle suspension was added to acetonitrile for demulsification. The propofol concentration in the aqueous phase of the mixed micelles was determined by ultracentrifugation at 3500× *g* for 40 min by an ultracentrifuge centrifuge tube (Amicon ultracel 3.5 K, Millipore, Billerica, MA, USA). The propofol concentration in micelles was measured by high performance liquid chromatography (HPLC), respectively. Agilent series HPLC system was equipped with a UV detector (Agilent 1260) and C_18_ column (250 mm × 4.6 mm, 5 μm), (Dikma Technologies, Beijing, China). Acetonitrile and water with 0.4% phosphoric acid were applied as the mobile phase. The gradient elution program is shown in Table 1. The flow rate was maintained at 1.0 mL/min with a column temperature of 35 °C. The injection volume was 20 μL and the detection wavelength was 270 nm. The EE of the propofol was calculated using the following formula:

$$\mathrm{EE}\left(\%\right)=\frac{M_{a}-M_{b}}{M_{c}}$$

where M_a_ is the total content of propofol in the micellar solution, M_b_ if the content of free propofol in the micellar solution, and M_c_ is the initial mass of propofol used in micelles.

To determine the drug loading content (LC) of propofol, the mixed micelles were lyophilized and dissolved into acetonitrile. Then, the propofol content was detected by HPLC analysis according to the above method. The LC of propofol was then calculated using the following formula:

$$\mathrm{LC}\left(\%\right)=\frac{M_{d}-M_{e}}{M_{f}}$$

where M_d_ is the total content of propofol in freeze-dried micelles, M_e_ the content of free propofol in freeze-dried micelles, and M_f_ the total mass of freeze-dried micelles.

### 2.5. Particle Size and Zeta Potential

The particle diameter, polymer dispersity index (PDI), and zeta potential of propofol formulation were measured with a dynamic light scattering (DLS) technique (Zetasizer Nano-ZS, Malvern, UK).

### 2.6. Morphology

The morphologies of propofol-mixed micelles were visualized by a transmission electron microscopy (TEM) (Tecnai^TM^ G2 spirit BioTWIN, Hillsboro). The propofol-mixed micelles were deposited onto the copper net by dropping droplets. Three minutes later, a drop of 1% (*w*/*v*) uranyl acetate was added to the copper grid and diluted negatively stained for three minutes. The samples were air-dried and the grid was observed in the TEM.

### 2.7. pH and Osmolarity

The pH of the propofol-mixed micelles and Diprivan was detected by a pH meter (PB-10, Sartorius Group, Goettingen, Germany). Their osmolarity was detected by a freezing point osmometer (Osmomat 030, Gonotec GmbH, Berlin, Germany).

### 2.8. Formulation Optimization

Box–Behnken design (BBD) was used to optimize the formulation. The design is suitable for exploring quadratic response surfaces and constructing second order polynomial models [26]. DSPE mPEG2k concentration, Solutol HS 15 concentration, and propofol concentrations were found to play key roles in affecting the drug LC and EE in the micelles. Therefore, we chose BBD to systemically evaluate the effect of the three key variables on LC and EE of the prepared mixed micelles. The range of each factor was determined according to the results of preliminary experiments and the feasibility of preparing the mixed micelles at the extreme values. The details of the study design are shown in Table 2. The BBD experiments were comprising of three-factors and three-levels designed through Design-Expert Program 8.0.6 software. A total of 17 experiments were performed. The 3D response surface plots were used to determine the importance of the three factors and their interrelationship.

### 2.9. Concentration of Free Propofol

The free propofol concentration of the mixed micelles and Diprivan was determined via a reverse dialysis method [26]. In brief, a dialysis tube (Spectrum Laboratories Inc., Piscataway, NJ, USA) (interception molecular weight: 1000 Da) was filled with a 300 µL of glycerol solution (2.5% *w*/*v*) and maintained in 15 mL of mixed micelles or Diprivan for 24 h at room temperature in a thermostatic water bath box. After removal from the propofol formulation, 100 μL of glycerol solution with acetonitrile was diluted to 1 mL of total volume, and then the free propofol concentration was determined by the above-mentioned HPLC analysis.

### 2.10. Pain on Injection

Pain behavior after injecting propofol formulations was assessed by the SD rat paw-lick test [26]. Rats were randomized in four groups, each consisting of six rats. Each group was treated with either 10 mg/kg of a saline, 0.6% acetic acid solution, Diprivan, or propofol-mixed micelles into the right hind footpad. The onset and duration spent licking the injected hind paw of each rat were recorded for 10 min. The injection site pain is often instantaneous and cannot persist for extended periods of time.

### 2.11. Histological Evaluation

Twenty male BALB/c mice (20–25 g) were randomized into four groups, and each group was intravenously injected with a saline, 0.6% acetic acid solution, Diprivan or propofol-mixed micelles at a dose of 5 mg/kg. The presence of local inflammation or tissue damage caused by the propofol preparation was evaluated after intraperitoneal administration [27]. The hematoxylin and eosin staining was performed on the peritoneal membranes sections and visualized under a light microscope.

### 2.12. Hemolysis Test

The hemolytic effect of propofol-mixed micelles in red blood cells (RBC) was investigated by using freshly blood from SD rats. In brief, blood samples were collected via cardiac puncture and immediately transferred to heparin sodium containing tubes. The red blood cells were collected by centrifugation (2000 rpm for 10 min), washed by PBS solution, and then diluted to 1/10 of their volume with PBS for further use. A total of 0.8 mL of different dilution rates (0-, 5-, 10-, 20-fold) of micelles were added to 0.2 mL of RBC suspension and incubated for 2 h at 37 °C in a shaking water bath (50 rpm). The absorbance of supernatants was analyzed at 545 nm after centrifugation at 2500 rpm for 5 min. Purified water and PBS buffer-treated erythrocyte solution were studied as positive (100% lysis) and negative (0% lysis) control, respectively. The hemolysis rate was then calculated using the following formula:

$$\mathrm{Hemolysis rate}\left(\%\right)=\frac{\mathrm{Abs}\left(\mathrm{sample}\right)-\mathrm{Abs}\left(\mathrm{negative control}\right)}{\mathrm{Abs}\left(\mathrm{positive control}\right)-\mathrm{Abs}\left(\mathrm{negative control}\right)}$$

### 2.13. Sleep/Recovery Studies

Male SD rats were injected intravenously with the propofol-mixed micelles or Diprivan at a dose of propofol 10 mg/kg to evaluate recovery from anesthesia. The end of the injection was assigned as time zero. The rats were placed in supine positions, and the loss and recovery time of the righting reflex was monitored.

### 2.14. Pharmacokinetic Assessment

The SD rats were injected with propofol-mixed micelles and the Diprivan through the tail vein in the dose of 10 mg/kg for the in vivo pharmacokinetic study. A volume of 400 µL blood samples were drawn by retroorbital bleeding at 2, 5, 10, 20, 30, 40, 60, 90, and 120 min after injection, and collected in the heparinized tubes. The plasma samples were separated from blood at 3000 rpm for 10 min, whereafter, the collected blood samples (100 µL) were diluted with 200 µL acetonitrile. The mixtures were vortexed for 3 min and centrifuged with a speed of 13,000 rpm for five min. The supernatants were analyzed by the above-mentioned HPLC method with a sample volume of 20 µL. The pharmacokinetic parameters, including distribution half-life (t_1/2α_), elimination half-life (t_1/2β_), T_max_ (the time to reach the C_max_), C_max_ (maximum plasma concentration), drug clearance (CL), and the area under the curve (AUC_(0–120 min)_) were analyzed using the Drug and Statistics (DAS) version 2.0 software (Shanghai University of Traditional Chinese Medicine, Shanghai, China).

### 2.15. Statistical Analysis

All data are presented as mean ± SD. The differences between samples were evaluated using the Student’s *t*-test, with *p* < 0.05 considered statistical significance.

## 3. Results and Discussion

### 3.1. Optimization of the Preparation Technology

In drug formulation development, high EE and LC were able to ensure that adequate drug delivery to produce therapeutic effects. In the present study, BBD was used to optimize formulations of the propofol-mixed micelles. A total of 17 runs was performed to evaluate the effect of three crucial factors on EE and LC.

A series of single-factor experiments indicated that LC and EE had pronounced changes with varying concentrations of DSPE mPEG2k, Solutol HS 15, and propofol. Therefore, the three factors were conducted as optimization variables through BBD at three experimental levels. The concentration of Solutol HS 15 (labelled as X_1_) ranged from 5 to 20 mg/mL, the concentration of DSPE mPEG2k (labelled as X_2_) ranged from 1 to 20 mg/mL, and the concentration of propofol (labelled as X_3_) ranged from 0.2 to 5.0 mg/mL. EE (Y_1_) and LC (Y_2_) were used as dependent variables (responses). The experimental design and results are shown in Table 3.

Table 4 shows the results of the variance analysis for the two responses. According to the regression coefficient significant in the quadratic regression model, factors X_1_ (*p* < 0.0001), X_2_ (*p* < 0.0001), and X_3_ (*p* < 0.0001) were significant terms affecting LC. The interaction terms (X_1×2_, X_1×3_, and X_2×3_) were also significant, whereas the quadratic terms (X_3_^2^ and X_2_^2^) were not significant (*p* > 0.05), and X_1_^2^ was significant for LC responses. For EE, X_1_ was the most significant factor (*p* < 0.0001), followed by X_2_ (*p* < 0.01). The interaction terms (X_1×2_, X_1×3_, and X_2×3_) were not significant (*p* > 0.05), whereas the quadratic terms (X_3_^2^ and X_2_^2^) were significant (*p* < 0.05), and X_1_^2^ was not significant for EE responses.

**Figure 2:**
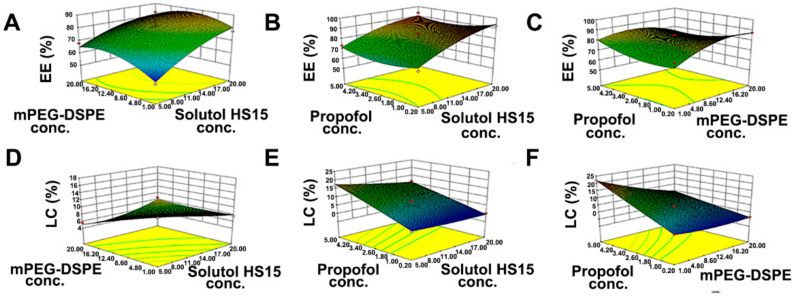
The 3D response surface plots diagrams of EE (**A**–**C**) and LC (**D**–**F**). X_1_, X_2_, and X_3_ represent the concentration of Solutol HS 15, DSPE mPEG2k, and propofol, respectively.

The 3D response surface plots were further used to estimate the combined effects of factors on responses (Figure 2). The application of statistical tools of response surfaces allowed determining the optimum experimental conditions for preparation of micelles with high EE and LC: a Solutol HS 15 concentration of 15.8 mg/mL, an DSPE mPEG2k concentration of 1.0 mg/mL, and a propofol concentration of 5.0 mg/mL. The optimized formulation was prepared and the resultant experimental results were compared with predicted values to verify the feasibility of the optimization process. The predicted values of EE and LC in the calculated model were 84.82% and 19.24%, respectively, and the resultant experimental values were close to the predicted values and percentage prediction error was less than 5% (Table 5). Thus, the BBD for optimization of propofol-mixed micelles was validated.

### 3.2. Particle Size and Zeta Potential

**Figure 3:**
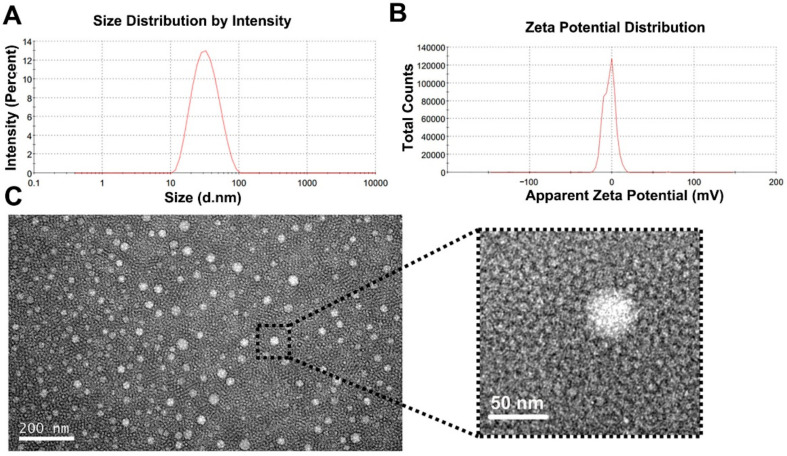
Characterization of the propofol-mixed micelles. (**A**) Size distribution, (**B**) zeta potential, and (**C**) representative TEM image.

As depicted in Figure 3, the propofol-mixed micelles show that that particle size was 29.9 nm and had a PDI of 0.163 (Figure 3A), which means its suitability for parenteral administration. The zeta potentials of −3.1 mV (Figure 3B) implied good stability of optimized formulation. The TEM image revealed that the propofol-mixed micelle had a spherical appearance (Figure 3C) and the diameters of the micelle particles were consistent with the finding above.

### 3.3. pH and Osmolarity

Unphysiological pH is one of key factors likely to induce injection site pain [28]. In our study, the detected pH of propofol-mixed micelles was 7.27 ± 0.02, which was slightly lower than the Diprivan (7.42 ± 0.03). However, it was within the suitable range, which was applicable to intravenous use. Similarly, the unphysiological osmotic pressure of the injectable formulation can also result in intravenous injection pain and, thus, it is necessary for determining the appropriate osmotic pressure of the parenteral formulation. The osmolarity of propofol-mixed micelles and Diprivan were 307.0 ± 1.7 mOsmol/L and 302.5 ± 1.1 mOsmol/L, respectively. Hence, propofol-mixed micelles showed acceptability for intravenous use.

### 3.4. Concentration of Free Propofol

Many factors induce injection-site pain associated with propofol, including the injection site, the vein size, the injection speed, and concentration of free propofol. However, the leading cause injection pain is related to free propofol concentration in the aqueous phase. Injection pain of propofol can be immediate or lagged reaction [29]. The immediate pain could be due to an irritant effect, while delayed pain possibly contributes to indirect impacts through the kinin cascade that has pain response latency. In clinical anesthesia, propofol is commonly used in combination with several drugs, such as lidocaine and sufentanil. These drugs have been successfully used to minimize propofol-induced pain by inhibiting pain transmission via free nerve endings of vessels, but without decreasing the free propofol concentration [30].

**Figure 4:**
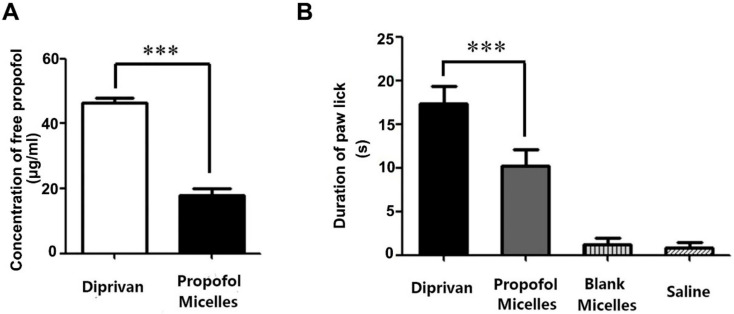
(**A**) Free propofol concentration in the aqueous phase of the mixed micelles and Diprivan (mean ± SD, *n* = 6). (**B**) Duration of rat paw-lick for propofol-mixed micelles and Diprivan (*n* = 6; *** *p* < 0.001).

In this study, we found that Diprivan contained a free propofol concentration of 46.2 ± 2.0 μg/mL in the aqueous phase. In comparison to the Diprivan, optimized propofol-mixed micelles exhibited a marked decrease in the free propofol concentration of 60.9% (*p* < 0.001) (Figure 4A). We speculate that the propofol-mixed micelles could induce less injection pain than marketed Diprivan.

### 3.5. Pain on Injection

The rat paw-lick study revealed that the propofol-mixed micelles have significantly less (*p* < 0.001) injection pain (10.17 ± 4.58 s) as compared to the marketed Diprivan (17.33 ± 4.88 s), as shown in Figure 4B. In addition, the results showed a similar effect in the blank mixed-micelles and the saline solution, meaning DSPE mPEG2k and Solutol HS 15 cannot produce any injection pain. Therefore, the less injection pain effect with propofol-mixed micelles could be due to less free propofol in the formulation.

### 3.6. Histological Evaluation

**Figure 5:**
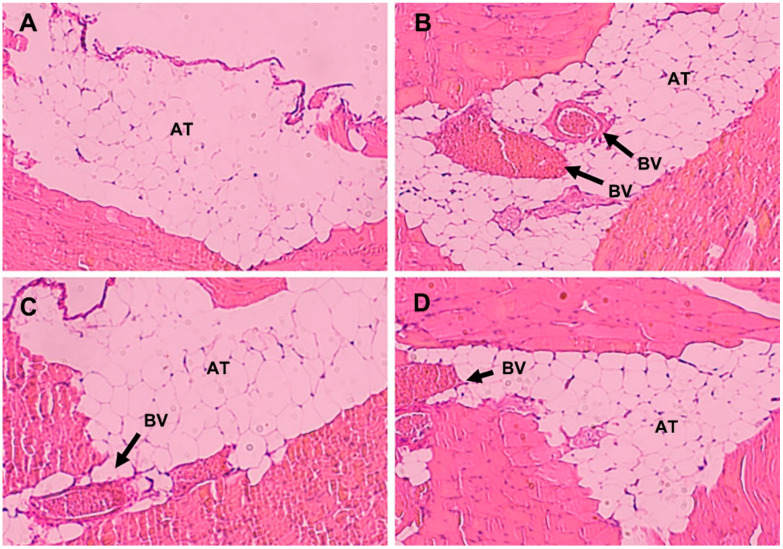
Representative HE-staining micrographs of peritoneal tissues in mice after intraperitoneal administration. Injection with saline (**A**), acetic acid (**B**), Diprivan (**C**), and propofol micelles (**D**). Arrows in the panels indicate AT = adipose tissue and BV = blood vessel.

The micrograph analysis shows no significant peritoneal inflammatory response observed after intraperitoneal injection of either propofol formulations (Figure 5). However, evident congestion of blood vessels is observed after intraperitoneal administration of acetic acid. It indicates that neither propofol formulation resulted in local tissue lesions or inflammation.

### 3.7. Hemolysis Test

**Figure 6:**
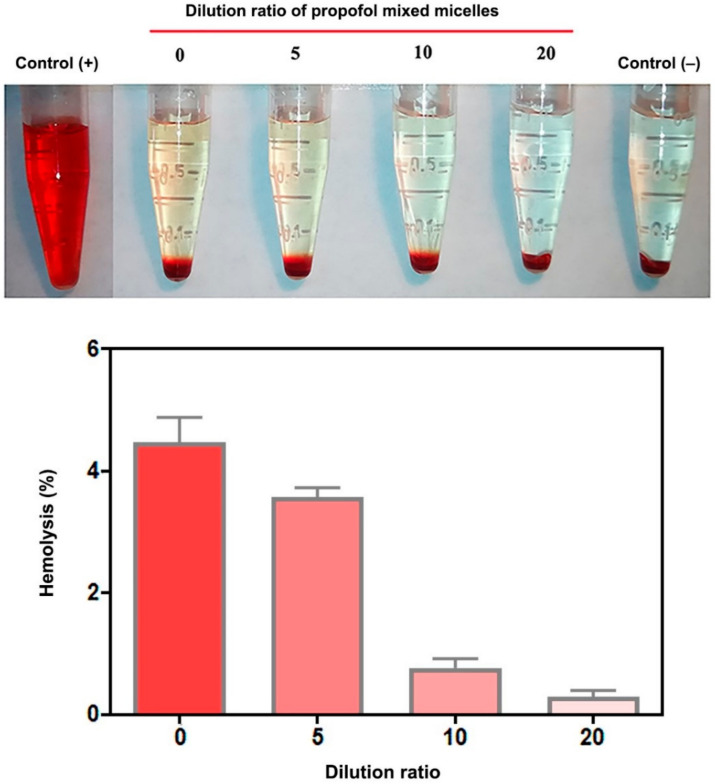
Hemolysis test results of the propofol-mixed micelles at different dilution ratios (*n* = 3).

It was reported that lipid emulsion Diprivan made with lecithin exhibited potential hemolytic activity, probably associated with the presence of lysophosphatidylcholine and phosphatidyl ethanolamine, which were produced by the hydrolysis of lecithin during preparation and storage of the formations [31]. Thus, to assess the blood compatibility, a hemolysis test of propofol-mixed micelles at different dilution ratios was conducted to confirm the biocompatibility. Figure 6 shows that the hemolysis rate of propofol-mixed micelles was lesser than 5%, indicating the micelles had a non-hemolytic reaction.

### 3.8. Anesthetic Action

**Figure 7:**
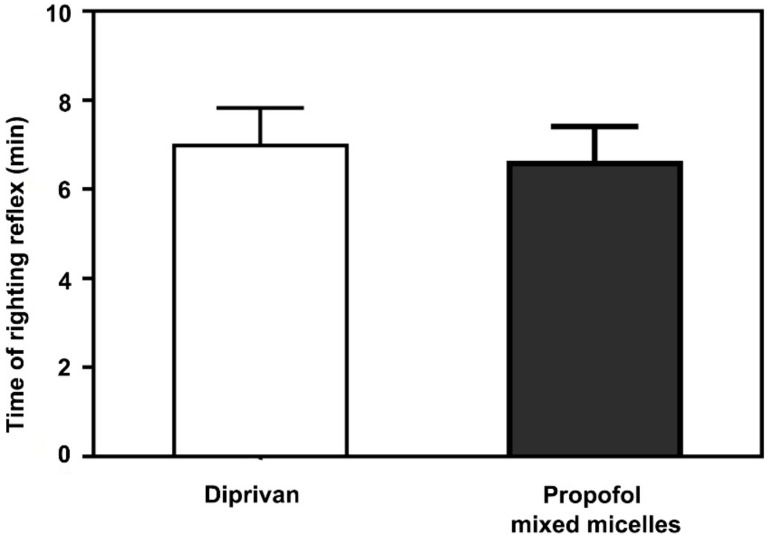
In vivo anesthetic action of propofol-mixed micelles and Diprivan (*n* = 9).

The average time for return of the righting reflex was recorded after commercial formulation (Diprivan) and propofol-mixed micelle administration. The time average of loss of the righting reflex of Diprivan and the different doses of micelle are shown in Figure 7. Following administration of the propofol-mixed micelles and Diprivan, animals rapidly lost motility within 20 s. The anesthetic action study of propofol formulation revealed that the propofol-mixed micelle at 10 mg/kg dose had a slightly longer time of anesthesia (8.05 ± 1.84 min) in comparison with the Diprivan (7.51 ± 1.74 min). The duration for the rats to lose and regain motility were not significantly different in both propofol formulations (*p* > 0.05). It could be deduced that the differences of both formulations in drug-release behavior did not substantially change the assignment of propofol in the central nervous systems of the rats. Therefore, similar pharmacological phenomena were presented.

### 3.9. Pharmacokinetic Study

**Figure 8:**
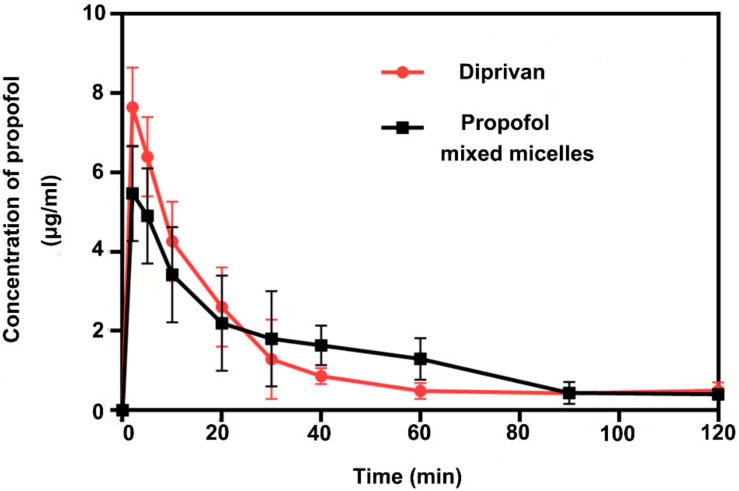
Plasma concentration of propofol vs. time for propofol-mixed micelles and Diprivan following intravenous administration (means ± SD, *n* = 5).

The average plasma concentration versus time profiles of the propofol-mixed micelles and Diprivan are displayed in Figure 8. The pharmacokinetic parameters were calculated by two-compartment modeling (Table 6). Initial plasma concentration of the propofol for the mixed micelles showed a slight decrease. The distribution half-life (t_1/2α_) of micelles was 6 min, which was approximately 40% shorter than that for Diprivan (10 min). Furthermore, the mixed micelles showed a shorter elimination half-life (t_1/2β_) than that of Diprivan. It might be due to the micellar-controlled release property and the drug needs more time to release from the system. In addition, similar results were observed in the apparent volume of CL and AUC_(0–120 min)_ between the propofol-mixed micelles and Diprivan, which means that the two formulations have similar absorption and clearance effects after a single dose. Table 6 shows that propofol was absorbed rapidly and eliminated quickly.

## 4. Conclusions

In summary, we successfully prepared propofol-mixed micelles using DSPE mPEG2k and Solutol HS 15. The mixed micelles showed homogenous particle sizes with diameters maintained at around 30 nm. The micelles were “low lipid”, which could diminish the frequency of hyperlipidemia, and the low concentration of free propofol significantly reduced pain in the rat paw-lick study compared to the Diprivan; thus, overcoming the major defect of the commercial formulation. More importantly, the micelle formulation displayed similar anesthetic actions, absorption, and clearance effects after a single dose in comparison with the marketed formulation. In addition, the novel propofol formulation had a non-hemolytic reaction and exhibited a good safety profile. Hence, the novel propofol formulation could act as a commercially viable formulation for parenteral injections of propofol and as a more valid alternative to Diprivan.

## Figures and Tables

**Table 1:** Gradient program for separation of the propofol.

Time (Min)	Water with 0.4% Phosphoric Acid (%)	Acetonitrile (%)
0	50	50
6	30	70
13	30	70
14	50	50
16	50	50

**Table 2:** Variables employed in BBD.

Independent Variable/Factor	Level		
	−1	0	1
X_1_:	5	12.5	20
X_2_:	1	10.5	20
X_3_:	0.2	2.6	5.0
Dependent variable/response	Constraints		
Y_1_:	Maximize		
Y_2_:	Maximize		

X_1_, X_2_, and X_3_ represent the concentration of Solutol HS 15, DSPE mPEG2k, and propofol, respectively. Y_1_ and Y_2_ are EE and LC, respectively.

**Table 3:** Experimental runs and results of responses for BBD.

Formulation Run	Factor 1 X_1_	Factor 2 X_2_	Factor 3 X_3_	Response 1 Y_1_	Response 2 Y_2_
1	5	1	2.6	51.66	17.94
2	20	1	2.6	77.32	8.52
3	5	20	2.6	67.88	6.5
4	20	20	2.6	82.55	5.24
5	5	10.5	0.2	66.73	1.05
6	20	10.5	0.2	92.45	0.07
7	5	10.5	5.0	73.2	17.96
8	20	10.5	5.0	93.38	12.26
9	12.5	1	0.2	75.46	2.34
10	12.5	20	0.2	87.32	0.63
11	12.5	1	5.0	80.21	22.54
12	12.5	20	5.0	71.52	8.88
13	12.5	10.5	2.6	85.15	8.62
14	12.5	10.5	2.6	78.44	7.98
15	12.5	10.5	2.6	79.52	8.08
16	12.5	10.5	2.6	80.43	7.18
17	12.5	10.5	2.6	79.10	8.04

**Table 4:** Analysis of variance for BBD.

Source	Y_1_		Remarks	Y_2_		Remarks
	F Value	*p*-Value Prob > F		F Value	*p*-Value Prob > F	
Model	9.95	0.0031	Significant	206.69	<0.0001	Significant
X_1_	55.62	0.0001		334.44	<0.0001	
X_2_	9.95	0.0031		111.32	<0.0001	
X_3_	4.53	0.0707		1223.39	<0.0001	
X_1 × 2_	0.10	0.7615		49.19	0.0002	
X_1 × 3_	0.46	0.5198		105.50	<0.0001	
X_2 × 3_	1.81	0.2208		16.46	0.0048	
X_1_^2^	3.89	0.0890		14.15	0.0071	
X_2_^2^	6.32	0.0402		1.15	0.3195	
X_3_^2^	11.46	0.0117		5.24	0.0559	
Lack of Fit	5.91	0.0453	Not significant	10.67	0.0223	Not Significant

**Table 5:** Predicted and experimental values for the optimized formulation.

Response	Predicted Value	Actual Value	Deviation
EE (%)	84.82	81.73 ± 0.65	3.64%
LC (%)	19.24	18.46 ± 0.82	4.95%

**Table 6:** Pharmacokinetic parameters of propofol following intravenous injection of propofol-mixed micelles and Diprivan to rats.

Parameter	Unit	Diprivan	Propofol-Mixed Micelles
t***_1/2_***_α_	min	10	6
t***_1/2_***_β_	min	69	384
T_max_	min	2	2
C_max_	μg/L	8	5
AUC (0–120 min)	μg·min/L	158	174
CL	L/min/kg	48	48

T_max_, (the time to reach the C_max_); C_max_, (maximum plasma concentration).

## Data Availability

Not applicable.
